# Hospital Facts for an Interested Public

**Published:** 1914-05-16

**Authors:** 


### HOSPITAL FACTS FOR AN INTERESTED PUBLIC.

**(Contributions for this Section Cordially Invited.)**

Our attention is frequently called to the wish expressed all over the country by an increasing number of people with means who desire terse but interesting facts illustrating phases in the daily work of our voluntary hospitals. Some of the most touching, amusing, and telling of stories, some of the most graphic and humanising incidents of human existence, some of the kindest and most helpful acts of the healthy to the dependent, illuminate hospital life. It would be an immense gain in credit and cash to each voluntary hospital if it possessed one active and interested person with the glad eye to write tersely and well the moving incidents of their hospital of each day or week. By way of illustration we publish a few experiences, given with telling effect to a great League of Mercy meeting at Wandsworth, by Mr. W. McAdam Eccles, to whom we are indebted for them at our request. We hope other members of the profession, and some of the laity, will lend a hand, now and again, by contributing hospital facts for insertion in this section.

By W. McADAM ECCLES, M.S., F.R.C.S., of St. Bartholomew's Hospital.*

Your Royal Highness, your Worship, my Lord, Ladies and Gentlemen,—It is a characteristic of English folk to be practical, and we are always desirous of knowing how the money which we subscribe to such a charity as a hospital is expended. It will be my duty to-night to indicate as briefly as I can some of the facts which tend to show how your contributions to the great London hospitals are used. Let me give you some idea by an illustration.

### With the Surgeon on Night Duty

When I had finished operating upon two emergency operations the other night when on surgical duty, the porter came up and said that a message had just come over the telephone to say that a doctor was sending up a little child with acute appendicitis, believing that it required an immediate operation in order to save its life. This telephone takes up some of your generous contributions, and is a factor in the saving of this child's life. Its message puts all the machinery of the hospital in action for the sake of the patient who is on the way. As soon as the taxi—which is often paid for by the kind doctor who has diagnosed and is sending the case—arrives, the gate-porter rings a bell, which announces the advent of the little sufferer, and the house-surgeon is quickly on the spot to receive her and confirm the doctor's opinion that the disease is a dire one, and operation, and that at once, the only remedy. The child is immediately transferred to a beautifully appointed ward, where the sister is still "on duty" although the hour is past midnight, and she comforts the mother, and tells her that the little one will soon be relieved. You cannot have a ward and its sympathetic sister and nurses without cost, and it is your money which has been helping to provide all this. Meanwhile the sister of the operation theatre and her assistants have been preparing for the reception of the patient there, and before long the child is taken to the theatre, where an anæsthetist is ready to give her the chloroform which is going to send her to sleep and rid her of all pain during the actual operation. Whilst the child is having the anæsthetic administered the surgeon and the house surgeon are preparing their hands and putting on the necessary overalls and rubber gloves, and the nurses have got ready the instruments and the many other articles which go to make up the requirements in the modern operation theatre. All of these cost money, and without your help this skilled preparation cannot be carried out.

### When a Hospital is Better than a Palace.

The hospital surgeon, who receives no fee for his labours, operates carefully but rapidly, and within half an hour the child is returned to a warm cot in the ward, and the mother is told of the success of the operation. But there is not yet an end to the beneficent work which is continued with the help of your money, for the child is nursed, fed, washed, and surgically treated in the ward of the voluntary hospital until she has quite recovered, and this may not be for ten days or longer. All this care and attention again implies cost, and the requisite money comes in part from you. I would even go so far as to say that this child, whose hospital history we have been tracing, has been receiving far better attention than many a child of richer parents, for when a child of the rich falls a victim to appendicitis, an operation is often undertaken in a private house, where there are often wanting many of the elaborate preparations and special appliances such as I have been outlining in my remarks.

### Some Interesting "Expenditure" Points.

I hold in my hand the statement of the expenditure of one of our large hospitals, and the items under the column giving the money spent on provisions exhibit some interesting points. I doubt whether many of you present could guess which article of diet has the largest amount of money expended upon it. I think some would venture to say meat, but it is not so. Milk stands first, and this cost no less than £2,370; whilst meat only cost £1,500. I turn over the page and I see an item of expenditure which will cause you to wonder—namely, £930 spent on "scrubbers."

* Abstract of an address given to the Kingston, Surbiton, Wandsworth, and Putney District of the League of Mercy.

What does this mean? It means that in a hospital cleanliness is essential for success, and cleanliness in London costs money, as most of you know who try to keep your houses clean. If an ordinary private house is difficult to keep clean, a hospital is even more so. We often wish that we could exclude the dirt which is brought into our clean wards by the "dirty" visitors who troop in twice a week! Of course we cannot say they must not come and see their sick folk, but great is the need for cleansing when they have departed, and often serious is the infection which they bring, and the disease which follows their sojourn, brief as it is, in our wards.

No, ladies and gentlemen, the wonder is that hospitals do not cost more than they do, not that they cost as much as they do. They are all exceedingly well administered, and you may be quite sure that the money which you so kindly subscribe is expended in the best possible manner, and is bringing the best possible results in the way of alleviation of suffering and the cure of disease to hundreds of thousands in our great Metropolis.

The Effect of the National Insurance Acts.

I would like to conclude with a word or two upon the effect of the National Health Insurance Acts upon the hospitals. I will put it very clearly, for it is an important matter. The number of those who apply for treatment as out-patients has diminished very appreciably; but these are the class of patients who cost the least. The number of in-patients has not diminished at all; in fact, the number who are applying for admission is now greater than before the introduction of the Acts, and is likely to increase rather than diminish. And this is the class of patient which costs money; in fact, some of them cost as much as £5 a week. **Further, the amount that the hospital to which I belong received during the whole of the twelve months of last year from the approved societies towards the maintenance and treatment of insured persons in the wards was the paltry sum of £86!**

Will the Public Please Remember?

So you can see that without the magnificent aid of the generous public these thousands of insured persons whom we admit into the wards of our numerous London hospitals would have to go without all the highly skilled medical and surgical treatment, and the splendid nursing, which latter is better in our islands than in any other part of the world. I think, therefore, Your Royal Highness, we are not too importunate in asking each one present, and, indeed, every Leaguer, to strive his or her utmost to do the practical work of making known the needs of the hospitals, and of getting them supplied in every possible way, and not the least in that excellent method of one shilling a month from every household of the huge city in which our lot is cast.

